# Estimation of the Percentage of US Patients With Cancer Who Are Eligible for and Respond to Checkpoint Inhibitor Immunotherapy Drugs

**DOI:** 10.1001/jamanetworkopen.2019.2535

**Published:** 2019-05-03

**Authors:** Alyson Haslam, Vinay Prasad

**Affiliations:** 1 Knight Cancer Institute, Oregon Health & Science University, Portland; 2 Division of Hematology Oncology, Knight Cancer Institute, Oregon Health & Science University, Portland; 3 Department of Public Health and Preventive Medicine, Oregon Health & Science University, Portland; 4 Center for Health Care Ethics, Oregon Health & Science University, Portland; 5 Division of General Medicine, Department of Medicine, Oregon Health & Science University, Portland

## Abstract

**Importance:**

Immunotherapy checkpoint inhibitors have generated considerable interest because of durable responses in a number of hitherto intractable tumor types.

**Objective:**

To estimate the percentage of patients with cancer in the United States who are eligible for and respond to checkpoint inhibitor drugs approved for oncology indications by the US Food and Drug Administration (FDA).

**Design, Setting, and Participants:**

Retrospective cross-sectional study performed from June 2018 through October 2018 using publicly available data to determine (1) demographic characteristics of patients with advanced or metastatic cancer, (2) FDA data on checkpoint inhibitors approved from January 2011 through August 2018, (3) measures of response from drug labels, and (4) published reports estimating the frequency of various inclusion criteria.

**Main Outcomes and Measures:**

The estimated percentages of US patients with cancer who are eligible for and who respond to immunotherapy checkpoint inhibitor drugs, by year.

**Results:**

Six checkpoint inhibitor drugs were approved for 14 indications between March 25, 2011, and August 17, 2018. The estimated percentage of patients with cancer who were eligible for checkpoint inhibitor drugs increased from 1.54% (95% CI, 1.51%-1.57%) in 2011 to 43.63% (95% CI, 43.51%-43.75%) in 2018. The percentage of patients with cancer estimated to respond to checkpoint inhibitor drugs was 0.14% (95% CI, 0.13%-0.15%) in 2011 when ipilimumab was approved for unresectable or metastatic melanoma and increased to 5.86% (95% CI, 5.80%-5.92%) by 2015. By 2018, the estimated percentage of responders increased to 12.46% (95% CI, 12.37%-12.54%).

**Conclusions and Relevance:**

The estimated percentages of patients who are eligible for and who respond to checkpoint inhibitor drugs are higher than reported estimates for drugs approved for genome-driven oncology but remain modest. Future research should explore biomarkers to maximize the benefit of immunotherapy among patients receiving it.

## Key Points

**Question:**

What is the estimated percentage of US patients with cancer who are eligible for and respond to checkpoint inhibitor drugs approved for oncology indications by the US Food and Drug Administration?

**Findings:**

This cross-sectional study found that the estimated percentage of US patients with cancer who are eligible for checkpoint inhibitor drugs increased from 1.54% in 2011 to 43.63% in 2018. The percentage of patients estimated to respond to checkpoint inhibitor drugs was 0.14% in 2011 and increased to 12.46% in 2018.

**Meaning:**

The estimated percentages of patients who are eligible for and who respond to checkpoint inhibitor drugs are higher than reported estimates for drugs approved for genome-driven oncology but remain modest.

## Teaser

This cross-sectional study estimates the percentage of US patients with cancer who were eligible for and who responded to checkpoint inhibitor immunotherapy drugs between 2011 and 2018.

## Introduction

Cancer checkpoint inhibitors have received considerable and broad interest because of their ability to generate durable responses in many hitherto intractable malignant tumors^1,2^ and for improvements in overall survival in several randomized trials. These promising drugs, and their underlying preclinical science, formed the basis of the 2018 Nobel Prize in Medicine.^3^

Checkpoint inhibitors currently approved by the US Food and Drug Administration (FDA) target the cytotoxic T-lymphocyte–associated protein 4 (CTLA-4), programmed cell death receptor 1 (PD-1), or programmed cell death ligand 1 (PD-L1) and work by preventing immune evasion from cancer cells. The first approved agent, ipilimumab, received FDA marketing authorization in 2011 for metastatic melanoma. Since then, 5 more checkpoint inhibitor drugs have been approved for a total of 14 different indications (eTable in the Supplement).

Between 2015 and 2017, the number of clinical trials using PD-1 and PD-L1 inhibitors has increased nearly 600%, from 215 trials to more than 1500.^4^ The market is expected to grow similarly, from $1 billion dollars in 2013 to $7 billion dollars in 2020.^5^ However, despite growing interest in checkpoint inhibitors,^6,7,8,9,10^ empirical analyses have quantified the use of these drugs only in certain tumor types.^11^ To our knowledge, there has been no empirical analysis of the potential use or benefit among all US patients with cancer. For this reason, we sought to estimate what percentage of US patients with cancer are eligible for checkpoint inhibitors and what percentage might respond to them. Our analysis of checkpoint inhibitors is similar to a prior analysis^12^ of the estimation of genome-driven cancer therapies in US patients with cancer, which estimated a benefit of less than 5%. We hypothesized that the benefit of response from checkpoint inhibitors will be modest.

## Methods

We sought to estimate the percentage of US patients with cancer who are (1) eligible for and (2) may respond to FDA-approved checkpoint inhibitor drugs in a cross-sectional analysis. We defined persons as eligible for checkpoint inhibitors if they had the tumor type and notable inclusion criteria of the drug approval (eg, PD-L1 level). We defined persons as responding to therapy based on the best available response rate (FDA drug label) for that indication. We report annual findings from 2011 to present. Our methods are comparable to a prior analysis of genome-driven cancer therapies.^12^ We have reported our study according to the Strengthening the Reporting of Observational Studies in Epidemiology (STROBE) reporting guideline for cross-sectional studies. Per Oregon Health & Science University policy, this study was not submitted for institutional review board approval because it did not involve health care records and because all data are publicly available. The study was conducted between June 2018 and October 2018.

### Data Set

We selected all checkpoint inhibitor oncology drugs that were approved by the FDA through August 17, 2018.^13^ We included all indications, even those that had conditional approval under the accelerated pathway. For each drug and year for which the drug was approved, the approved indication and the overall response rate (complete plus partial) were extracted from the FDA label.

We used the overall response rate in the experimental group if the drug was compared with a placebo control, a different drug control, or was assessed in a single-group trial. The difference in overall response was used if a trial was run with a treatment backbone given with or without the checkpoint inhibitor. Overall response rates that were from exploratory analysis were also not used (eg, nivolumab for hepatocellular carcinoma with a Modified Response Evaluation Criteria in Solid Tumors).

### Statistical Analysis

To determine the percentage of patients who could potentially be candidates for, and therefore benefit from, a checkpoint inhibitor drug, we used annual cancer deaths as a stand-in for annual incidence of advanced or metastatic disease. This was similar to our prior work.^12^ Notably, stage IV disease incidence could not be used, as this does not include relapsed, metastatic disease that initially presented as early stage.^14^

Data on cancer deaths were obtained from American Cancer Society publications on cancer statistics, published between 2011 and 2018, to correspond with years that checkpoint inhibitor drugs had approval(s). Death statistics on cancers were aggregated by overall type, and the drugs for which there were approvals were sometimes approved only for a subgroup of that cancer. For example, lung cancer was subdivided into non–small cell lung cancer (NSCLC) and small cell lung cancer (SCLC). The category of NSCLC could be further subdivided by PD-L1 expression. In this situation, the number of deaths from lung cancer was multiplied by 85% to get an estimate of eligible patients with NSCLC and by 15% to get an estimate of eligible patients with SCLC. To estimate the number of patients with PD-L1 expression greater than 50%, the NSCLC estimate was then multiplied by 25%.^15,16^ The estimate for patients with PD-L1 expression of 0% to 50% was then calculated by taking the difference between the NSCLC estimate and the estimate for PD-L1 greater than 50%. In the case of urothelial carcinoma, pembrolizumab was approved in 2017 but reported different objective response rates for people who had been previously treated and people who had urothelial cancer that was cisplatin ineligible. In this case, for the years 2017 and 2018, the estimated benefit was calculated separately for cisplatin-ineligible and cisplatin-independent objective response rates based on previously reported estimates of individuals with cisplatin-ineligible disease and then the estimated benefits were combined into a single urothelial cancer measure.^17^

For each cancer type, the number of people estimated to be eligible for therapy was multiplied by the overall response rate reported in the FDA drug label for each year the drug was approved for that indication. This provided an estimate of the cancer-specific benefit or, in other words, the number of people who could potentially benefit. In selecting the overall response rate, the highest response rate reported by each drug approved for the specific cancer was used, thus giving the most generous estimate of benefit. Similarly, if multiple drugs were approved for a certain indication, the response rate from the drug showing the highest benefit was used. For each year, the estimated cancer-specific benefit for all indications that had an FDA-approved drug were summed. The sum of the estimated cancer-specific benefits for each year was then divided by the total number of people who died during that year from cancers for which there was an FDA-approved checkpoint inhibitor drug to derive an overall estimate of responders.

To calculate an estimate of the percentage of people who were eligible for checkpoint inhibitors, the number of cancer-specific deaths for which there were FDA-approved checkpoint inhibitors was divided by the total number of cancer deaths. To provide context, a ratio was also calculated dividing the percentage benefit from immunotherapy checkpoint inhibitors by the percentage of cancers affected by immunotherapy drugs (percentage eligible) (eFigure 1 in the Supplement). This descriptive analysis was done in Excel (Microsoft Corp). Calculation of 95% confidence intervals for the percentage of patients who were eligible and who could benefit were done in the MASS package of R statistical software version 3.5.0 (R Project for Statistical Computing).

## Results

Six checkpoint inhibitor drugs were approved for 14 indications between March 25, 2011, and August 17, 2018. These drugs and their original FDA approval date (year) were ipilimumab (2011), nivolumab (2014), pembrolizumab (2014), atezolizumab (2016), avelumab (2017), and durvalulmab (2017). The drugs were approved for these indications: melanoma, hepatocellular carcinoma, SCLC, NSCLC, renal cell carcinoma, urothelial carcinoma, Hodgkin lymphoma, head and neck squamous cell carcinoma, Merkel cell carcinoma, microsatellite instability–high colorectal cancer, gastric cancer, microsatellite instability–high cancers (noncolorectal), primary mediastinal large B-cell lymphoma, and cervical cancer.

### Eligible for Immunotherapy

**Figure 1.**
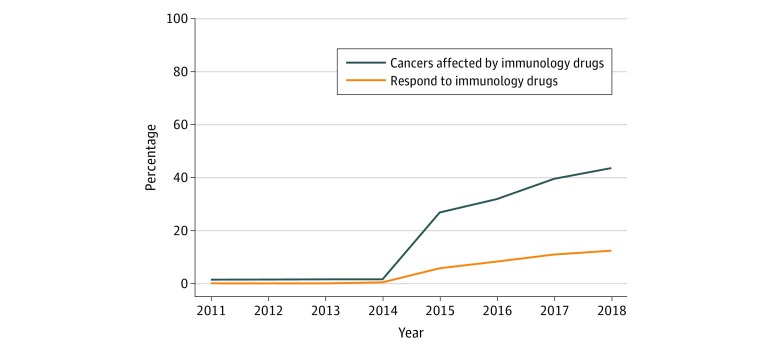
Percentage of US Patients With Cancer Who May Benefit From and Respond to Checkpoint Inhibitor Immunology Drugs (2011-2018)

**Figure 2.**
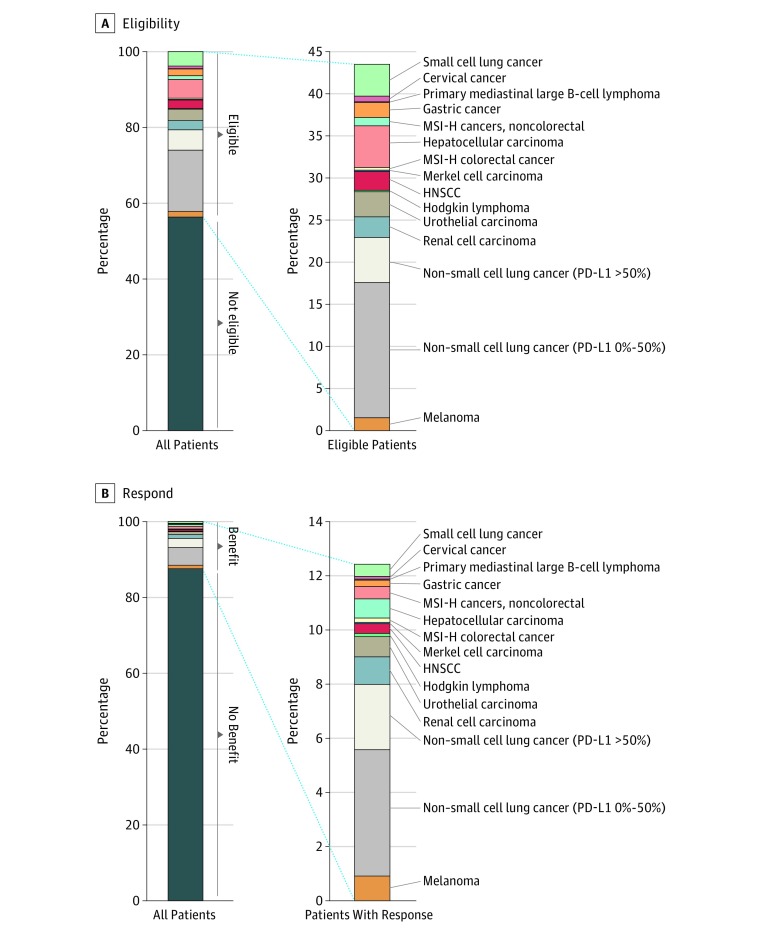
Percentage of US Patients With Cancer Eligible to Receive Checkpoint Inhibitor Drugs and Percentage Who Respond, by Cancer Type, in 2018 HNSCC indicates head and neck squamous cell carcinoma; MSI-H, microsatellite instability–high; and PD-L1, programmed cell death ligand 1.

In 2011, the total number of cancer deaths was 571 950; in 2015, it was 589 430; and in 2018, it was 609 640. The estimated percentage of patients in the United States with cancer eligible for checkpoint inhibitor drugs was 1.54% (95% CI, 1.51%-1.57%) in 2011 and increased to an estimated 26.86% (95% CI, 26.75%-26.98%) by 2015 and 43.63% (95% CI, 43.51%-43.75%) in 2018 (Figure 1). As of 2018, the indications that contributed most to the eligibility estimate included NSCLC (21.48%), hepatocellular carcinoma (4.95%), and SCLC (3.79%) (Figure 2).

### Responding to Immunotherapy

The estimated percentage of responders to checkpoint inhibitor drugs was 0.14% (95% CI, 0.13%-0.15%) in 2011, when ipilimumab was approved for unresectable or metastatic melanoma (Figure 1). The estimated number of responders slowly increased until 2015, when nivolumab and pembrolizumab were approved for NSCLC, at which time it was 5.86% (95% CI, 5.80%-5.92%). Since then, the estimate steadily increased to 12.46% (95% CI, 12.37%-12.54%) in 2018. As of 2018, the indications that contributed most to the response estimate included NSCLC (7.09%), renal cell carcinoma (1.02%), and melanoma (0.92%) (Figure 2).

The Table and eFigure 1 in the Supplement show the percentage benefit of checkpoint inhibitors for the specific cancer. Melanoma, the first condition for which a checkpoint inhibitor was approved, potentially resulted in responses in 0.14% of all patients with cancer during 2011, the year that it was first approved. Estimated responders increased to 0.53% when a PD-1 drug was approved for this indication. Responders increased again in 2015 to 1.01%, when higher response rates were reported in patients with *BRAF* V600 mutations, and the benefit has leveled off since then. Urothelial carcinomas have also seen a noticeable increase in the estimated number of patients who respond, with the percentage increasing from 0.43% in 2016 to 0.75% in 2018 after a checkpoint inhibitor drug was approved in 2014 for use in patients ineligible for cisplatin treatment. Response for renal cell carcinoma was somewhat stable from 2015 to 2017 (0.51% for all years) but increased to 1.02% in 2018 with the approval of a different checkpoint inhibitor drug combination that reported a higher response rate. In 2015, with the approval of drugs for NSCLC, it was estimated that 4.33% of cancers would benefit from a checkpoint inhibitor drug. Most of the benefit for checkpoint inhibitor drugs after 2015 was due to the responders with NSCLC: 5.92% in 2016, 6.78% in 2017, and 7.09% in 2018, although the benefit of checkpoint inhibitors for NSCLC was a lower proportion in later years than in earlier years, with the approval of multiple checkpoint inhibitors for more indications.

**Table.** Eligibility and Benefit for All Cancers and Benefit for Specific Cancers From Checkpoint Inhibitor Drugs From 2011 to 2018

Eligibility or Benefit	2011	2012	2013	2014	2015	2016	2017	2018
All cancers, % (95% CI)								
Eligibility	1.54 (1.51-1.57)	1.59 (1.56-1.62)	1.63 (1.60-1.67)	1.66 (1.62-1.69)	26.86 (26.75-26.98)	31.95 (31.84-32.07)	39.54 (39.41-39.66)	43.63 (43.51-43.75)
Benefit	0.14 (0.13-0.15)	0.15 (0.14-0.16)	0.15 (0.14-0.16)	0.53 (0.51-0.55)	5.86 (5.80-5.92)	8.36 (8.29-8.43)	11.04 (10.96-11.12)	12.46 (12.37-12.54)
Cancer-specific benefit, % (95% CI)								
Melanoma	0.14 (0.13-0.15)	0.15 (0.14-0.16)	0.15 (0.14-0.16)	0.53 (0.51-0.55)	1.01 (0.99-1.03)	1.02 (0.99-1.04)	0.97 (0.95-1.00)	0.92 (0.89-0.94)
Non–small cell lung cancer	NA	NA	NA	NA	4.33 (4.28-4.38)			
Non–small cell lung cancer (PD-L1 0%-50%)	NA	NA	NA	NA	NA	3.38 (3.34-3.43)	4.30 (4.26-4.37)	4.67 (4.62-4.73)
Non–small cell lung cancer (PD-L1 >50%)	NA	NA	NA	NA	NA	2.54 (2.50-2.58)	2.48 (2.44-2.52)	2.42 (2.38-2.45)
Renal cell carcinoma	NA	NA	NA	NA	0.51 (0.50-0.53)	0.51 (0.50-0.53)	0.52 (0.50-0.53)	1.02 (1.00-1.05)
Urothelial carcinoma	NA	NA	NA	NA	NA	0.43 (0.41-0.45)	0.74 (0.71-0.77)	0.75 (0.72-0.78)
Hodgkin lymphoma	NA	NA	NA	NA	NA	0.12 (0.11-0.13)	0.12 (0.11-0.13)	0.12 (0.11-0.13)
Head and neck squamous cell carcinoma	NA	NA	NA	NA	NA	0.35 (0.34-0.37)	0.36 (0.34-0.37)	0.36 (0.34-0.38)
Merkel cell carcinoma	NA	NA	NA	NA	NA	NA	0.05 (0.04-0.05)	0.05 (0.04-0.05)
Microsatellite instability–high colorectal cancer	NA	NA	NA	NA	NA	NA	0.12 (0.11-0.13)	0.16 (0.15-0.17)
Hepatocellular carcinoma	NA	NA	NA	NA	NA	NA	0.69 (0.67-0.71)	0.71 (0.69-0.73)
Microsatellite instability–high noncolorectal cancer	NA	NA	NA	NA	NA	NA	0.46 (0.44-0.48)	0.46 (0.44-0.48)
Gastric	NA	NA	NA	NA	NA	NA	0.24 (0.23-0.26)	0.24 (0.22-0.25)
Primary mediastinal large B-cell lymphoma	NA	NA	NA	NA	NA	NA	NA	0.04 (0.03-0.04)
Cervical cancer	NA	NA	NA	NA	NA	NA	NA	0.10 (0.09-0.10)
Small cell lung cancer	NA	NA	NA	NA	NA	NA	NA	0.45 (0.44-0.47)

Abbreviations: NA, not applicable; PD-L1, programmed cell death ligand 1.

The ratio of percentage benefit to percentage of cancers with FDA-approved checkpoint inhibitor drugs was 0.09 in 2011, peaked in 2014 at 0.32, and in 2018 was estimated to be 0.28 (eFigure 2 in the Supplement).

## Discussion

Checkpoint inhibitor drugs have generated deserved excitement in the field of oncology and are enjoying rapid uptake.^11^ Here, we present upper bound estimations of the percentage of US patients with cancer eligible for and responding to these drugs based on publicly collected and available data, assuming universal access to these medications.

The results of our analysis suggest that checkpoint inhibitors may at best lead to responses among less than 13% of patients with cancer in the United States. While the estimated percentage of people who respond to checkpoint inhibitor immunotherapy drugs is small, the benefit may be greater than some other drug classes in oncology owing to reports of durability. Recently, genome-targeted therapy, which has also generated considerable excitement, was estimated to benefit only 4.9% of patients with cancer.^12^

For the first few years after checkpoint inhibitors were initially approved for any oncology indication, the percentage of eligible patients was small and curves remained flat. In 2015, checkpoint inhibitors gained FDA approval for NSCLC, leading to a noticeable increase in benefit. As of 2015, only 3 checkpoint inhibitors were approved for 3 cancers, but since then, 3 other drugs have been approved, and the number of cancers for which these drugs have been approved has grown to 14, including the most recent approval of nivolumab for SCLC. However, even though the number of indications has increased substantially in recent years, the increase in benefit from these drugs in terms of the percentage of patients responding has slowed. Moreover, the percentage of individuals who are eligible to receive these drugs has grown since 2014 at a faster rate than the estimated percentage of individuals who actually benefit from these drugs.

One striking observation from the figures presented here is that the overall estimated benefit is driven mainly from NSCLC checkpoint inhibitor drugs, for which the benefit is somewhere around 7% of cancer deaths. This can be seen in Figure 2B, where the percentage of benefit is much larger for NSCLC than for other indications. This finding suggests that large improvements in US cancer statistics may be driven by drugs that are active in the most common tumor types.

The higher cancer-specific benefit in the group of patients with PD-L1 from 0% to 50% may seem counterintuitive at first glance, given that the response rate for NSCLC with PD-L1 greater than 50% is much higher than for NSCLC with PD-L1 between 0% and 50%. However, we estimated that there were approximately 3 times as many people who died from NSCLC with PD-L1 between 0% and 50% than NSCLC with PD-L1 greater than 50% during 2018, and the absolute numbers of individuals who develop and die from NSCLC with PD-L1 between 0% and 50% is what is driving the benefit.

For these analyses, we did include drugs that had conditional approval, such as nivolumab for hepatocellular carcinoma and other types of cancers. There is the possibility that these drugs fail to show benefit in confirmatory studies; in fact, pembrolizumab was conditionally approved for hepatocellular carcinoma but recently failed to improve more salient outcomes, such as overall survival, in a confirmatory study.^18^ We acknowledge that including these drugs may bias the results toward more favorable estimates, but once a drug is approved, it continues to be prescribed and is rarely withdrawn from the market.^19^ As such, these drugs should be included this type of analysis.

### Limitations

There are several limitations to our analysis. First, our study estimates but does not measure the uptake of these drugs. We make a number of favorable assumptions, such as using the highest response rate and assuming immediate and perfect access; thus, our estimate may be seen as upper bound, akin to prior work on genomic therapy. Furthermore, these estimates are based on response rates from clinical trials, which often include younger patients with fewer comorbidities.^20,21^ Further research should be done to clarify the effectiveness of these drugs in the real world.

Second, we did not consider off-label use in our analysis. Off-label use of checkpoint inhibitor drugs can be notable, with estimates of between 18% and 30%, depending on the drug and study.^22,23^ Popular articles have described the use of “desperation oncology,” in which immunotherapy is used to treat a variety of malignant neoplasms in patients nearing death.^24,25^ Estimating off-label use is difficult, as there is no reliable registry showing how many patients are exposed to therapy and what percentage respond.

Third, death from a specific cancer was used as a surrogate for the number of people eligible to benefit from a checkpoint inhibitor drug. This seemed reasonable to us, as most checkpoint inhibitors are approved for indications in the second line or later, and patients to whom they would be prescribed have a generally poor prognosis.

## Conclusions

If FDA-approved checkpoint inhibitor drugs are universally available, we estimated that the proportion of US patients with cancer who could be eligible for such drugs is approximately 44%, while approximately 13% have a response to these drugs. These estimates, although modest, are better than estimates for oncology drugs in other classes, such as genome-targeted therapies. These results may help policy makers, journalists, and physicians have more realistic discussions about checkpoint inhibitor drugs. Moreover, we hope these results will motivate researchers to develop drugs that benefit an even larger percentage of individuals with cancer than these current estimates.

## Notes

Supplement. **eTable.** Checkpoint Inhibitors Approved by the US Food and Drug Administration **eFigure 1.** Cumulative Percentage of Oncology Patients Who Are Eligible for and Who May Benefit From Checkpoint Inhibitor of Immunology Drugs, by Drug Type (2011-2018) **eFigure 2.** The Ratio of Patients in Oncology Who Respond to Checkpoint Inhibitors and Who Are Eligible for Them, During Years That Checkpoint Inhibitor Drugs Had Approvals for Oncology Indications
