# Supplementary material for: Estimation of the Percentage of US Patients With Cancer Who Are Eligible for and Respond to Checkpoint Inhibitor Immunotherapy Drugs
Source: JAMA Netw Open. 2019 May 3;2(5):e192535. doi: 10.1001/jamanetworkopen.2019.2535 (PMC6503493; doi:10.1001/jamanetworkopen.2019.2535)
Supplement: Supplement. — eTable. Checkpoint Inhibitors Approved by the US Food and Drug Administration eFigure 1. Cumulative Percentage of Oncology Patients Who Are Eligible for and Who May Benefit From Checkpoint Inhibitor of Immunology Drugs, by Drug Type (2011-2018) eFigure 2. The Ratio of Patients in Oncology Who Respond to Checkpoint Inhibitors and Who Are Eligible for Them, During Years That Checkpoint Inhibitor Drugs Had Approvals for Oncology Indications [file jamanetwopen-2-e192535-s001.pdf]

Supplementary Online Content

Haslam A, Prasad V. Estimation of the percentage of US patients with cancer who are eligible for and respond to checkpoint inhibitor immunotherapy drugs. *JAMA Netw Open*. 2019;2(5):e192535. doi:10.1001/jamanetworkopen.2019.2535

eTable. Checkpoint Inhibitors Approved by the US Food and Drug Administration

eFigure 1. Cumulative Percentage of Oncology Patients Who Are Eligible for and Who May Benefit From Checkpoint Inhibitor of Immunology Drugs, by Drug Type (2011-2018)

eFigure 2. The Ratio of Patients in Oncology Who Respond to Checkpoint Inhibitors and Who Are Eligible for Them, During Years That Checkpoint Inhibitor Drugs Had Approvals for Oncology Indications

This supplementary material has been provided by the authors to give readers additional information about their work.

© 2019 Haslam A et al. *JAMA Network Open.*

eTable: Checkpoint inhibitors approved by the US Food and Drug Administration

Drug	*Approval date*	*Tumor indications initially approved*	*Response rate (%) reported at initial approval*	*Approval type (conditional or full)*
Ipilimumab	*2011*	*melanoma*	*10.9*	*full*
	2018	*MSI-CRC*	*49*	*conditional*
	2018	*RCC*	*41.6*	*full*
Nivolumab	*2014*	*melanoma*	*32*	*conditional (initial)*
	2015	*NSCLC*	*19*	*full*
	2015	*RCC*	*21.5*	*full*
	2016	*Hodgkin's lymphoma*	*65*	*conditional*
	2016	*HNSCC*	*13.3*	*full*
	2017	*urothelial carcinoma*	*19.6*	*conditional*
	2017	*MSI-CRC*	*32*	*conditional*
	2017	*hepatocellular carcinoma*	*14.3*	*conditional*
	2018	*SCLC*	*12*	*conditional*
Pembrolizumab	*2014*	*melanoma*	*24*	*conditional (initial)*
	2015	*NSCLC*	*41 (PD-L1 status >50% only)*	*conditional (initial)*
	2016	*HNSCC*	*16*	*conditional*
	2017	*Hodgkin's lymphoma*	*69*	*conditional*
	2017	*urothelial carcinoma*	*21 (previously treated); 29 (cisplatin ineligible)*	*conditional (initial)*
	2017	*MSI*	*36 (colon); 46 (other)*	*conditional*
	2017	*gastric cancer*	*13.3*	*conditional*
	2018	*primary mediastinal large B-cell lymphoma*	*45*	*conditional*
	2018	*cervical cancer*	*14.3*	*conditional*

© 2019 Haslam A et al. *JAMA Network Open.*

Atezolizumab	*2016*	*urothelial carcinoma*	*14.8*	*conditional*
	2017	*NSCLC*	*15*	*full*
Avelumab	*2017*	*Merkel cell carcinoma*	*33*	*conditional*
	2017	*urothelial carcinoma*	*13.3*	*conditional*
Durvalumab	*2017*	*urothelial carcinoma*	*17*	*conditional*
	2018	*NSCLC*	*12*	*full*

MSI=microsatellite instability; CRC=colorectal cancer; RCC=renal cell carcinoma; NSCLC=non-small cell lung cancer; HNSCC=head and neck squamous cell carcinoma; SCLC=small cell lung cancer; ; PD-L1= Programmed death-ligand 1.

© 2019 Haslam A et al. *JAMA Network Open.*

eFigure 1: Cumulative percentage of oncology patients who are eligible for and who may benefit from checkpoint inhibitor of immunology drugs, by drug type (2011-2018)

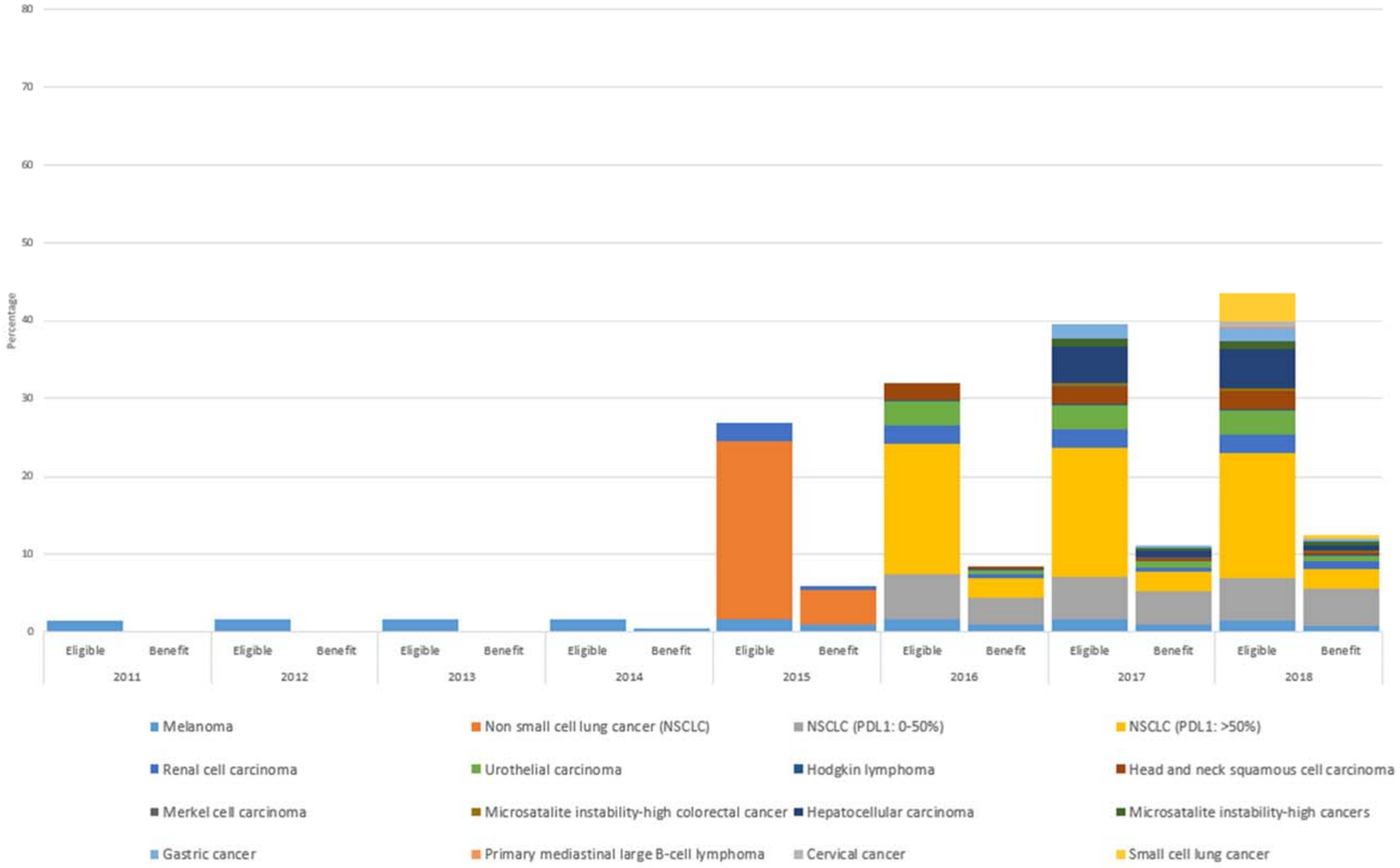

© 2019 Haslam A et al. *JAMA Network Open.*

eFigure 2: The ratio of patients in oncology who respond to checkpoint inhibitors and who are eligible for them, during years that checkpoint inhibitor drugs had approvals for oncology indications

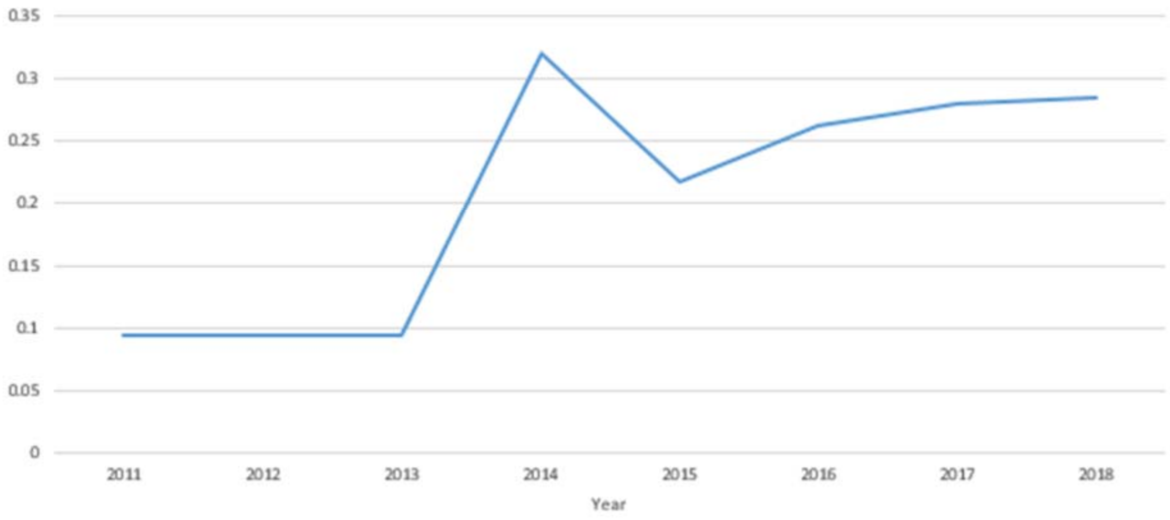

© 2019 Haslam A et al. *JAMA Network Open.*
